# Two Episodes of Ventriculoperitoneal Shunt Migration in a Patient with Idiopathic Intracranial Hypertension

**DOI:** 10.1155/2014/280793

**Published:** 2014-02-19

**Authors:** V. Balakrishnan, R. Jeanmonod

**Affiliations:** Department of Emergency Medicine, 618 Delaware Avenue, Bethlehem, PA 18015, USA

## Abstract

*Introduction*. Ventriculoperitoneal shunts are often placed as treatment for refractory idiopathic intracranial hypertension. Dislodgement and migration of the distal portion of the shunt are more common in obese patients and can be difficult to detect. We report the case of a woman with two separate episodes of shunt migration into her abdominal wall. *Case Presentation*. We report a case of a 37-year-old female with history of obesity eventually diagnosed with idiopathic intracranial hypertension (IIH) as the cause. She failed outpatient therapy and, through neurosurgery, had a VP shunt placed for symptom control. She had subsequent development of worsened symptoms that were found to be due to shunt migration. This happened not once but twice to the same patient. *Conclusion*. Shunt dislodgement, migration, and subsequent failure are common in obese patients who have shunts placed for IIH. The medical provider should maintain a high index of suspicion for shunt malfunction in these patients, particularly because clinical evaluation may be challenging due to habitus.

## 1. Introduction

Ventriculoperitoneal shunts are often placed as treatment for refractory idiopathic intracranial hypertension. Dislodgement and migration of the distal portion of the shunt are more common in obese patients and can be difficult to detect. We report the case of a woman with two separate episodes of shunt migration into her abdominal wall.

## 2. Case Presentation 

A 37-year-old female with a history of obesity presented to the emergency department (ED) multiple times over 1 year for the evaluation and treatment of headaches. She had poor control of these headaches despite appropriate medical management including acetazolamide, therapeutic lumbar punctures (LPs), and narcotics for the purported origin of pain being idiopathic intracranial hypertension (IIH) (formerly pseudotumor cerebri). Because of the chronicity of her visits to the ED and poor control of symptoms, neurosurgery made the decision to place a ventriculoperitoneal (VP) shunt.

**Figure 1:**
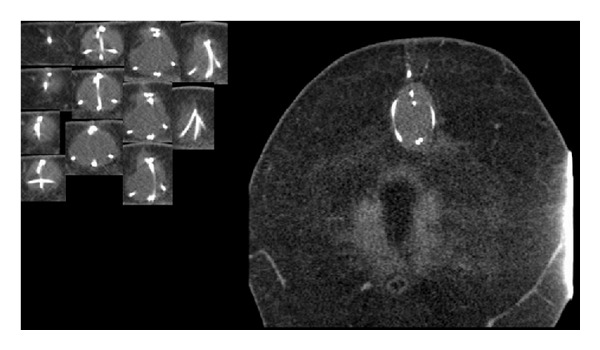
CT images from first VP shunt extrusion.

The surgery was successful, and postoperative radiographs confirmed appropriate position of the shunt. The patient was subsequently discharged home. Ten days after the procedure, the patient presented to the ED for a wound evaluation. She had noticed some swelling at the superior aspect of her abdominal wound. The patient reported that she had felt a “pop” in her abdomen while leaning over and straining to have a bowel movement a few days prior. Her exam was remarkable for mild induration and tenderness at her surgical incision. She was diagnosed with a seroma and discharged home. Her symptoms continued and she returned to the ED. A computed tomography (CT) of her abdomen/pelvis done at this visit demonstrated dislodgement of her VP shunt catheter and is shown in Figure 1.

The patient subsequently underwent externalization of this right-sided VP shunt followed by reinternalization a few days later without significant complications or abnormalities found during the procedure. She was then discharged home.

**Figure 2:**
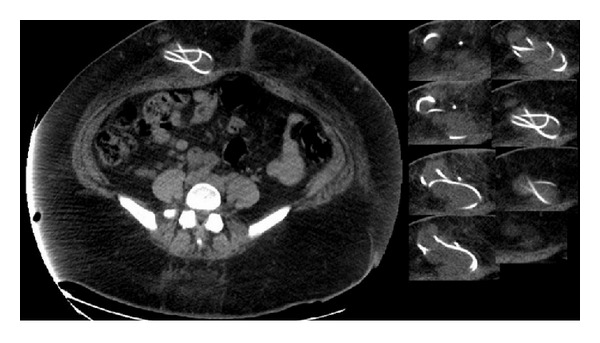
Second episode of VP shunt migration into the abdominal wall.

The patient represented to the ED two weeks later because of increasing headaches. She was managed symptomatically and underwent CT scanning of her head and shunt radiography, which were unremarkable. Her symptoms resolved and she was discharged home. One week later, the patient returned to the ED with abdominal pain which was steadily progressive. Her baseline headache was unchanged and she denied fever or neck pain. Her abdominal exam at that time revealed erythema and induration over the abdominal portion of her VP shunt. A CT showed redundant loops of the VP shunt in the right anterior abdominal wall (Figure 2).

The patient underwent a second externalization. Samples taken from the cerebrospinal fluid (CSF) pocket in the abdominal wall grew methicillin-resistant *Staphylococcus aureus* and *enterococcus*. Because of this infection, the right-sided shunt was completely removed. The patient had eventual resolution of her abdominal pain. Her IIH is now managed primarily by therapeutic LPs done under fluoroscopic guidance by interventional radiology.

## 3. Discussion

Idiopathic intracranial hypertension (IIH) is a rare condition, affecting about 0.9 patients per 100,000 in the US, with the majority of them being young women [1]. Although the etiology of IIH remains unclear, most adult patients with IIH are obese, and IIH has become more common as the rates of obesity have risen in the United States [2].

There is no true consensus for the treatment of patients with IIH. Experts agree that LP is both diagnostic as well as therapeutic, but repeated therapeutic LPs can be both painful as well as technically difficult in this population due to body habitus. Because the pathophysiology of the disease is unclear, the treatment strategy has been largely theoretical, with medical management primarily focusing on reducing CSF production and weight control and surgical interventions focusing on shunting CSF, reducing weight, or stenting cerebral vessels. VP shunting for refractory headache in IIH has been shown to be effective in the short term, but many patients require subsequent revisions and up to 20% may fail [3–6].

VP shunting in the obese patient can be particularly problematic. The reason for the failure of the VP shunt in the obese population is complex. Unlike patients with normal body mass index (BMI), obese patients with VP shunts have a system that essentially involves shunting of CSF from one high-pressure system (intraventricular) to a second high-pressure system (intraperitoneal) [7]. During Valsalva (such as while straining to stool) intra-abdominal pressure markedly increases, and this phenomenon is more pronounced in obese patients, whose intra-abdominal pressure is higher than patients with normal BMI even at baseline [8–10].

This increased intra-abdominal pressure can itself cause the VP shunt to be unable to drain CSF adequately, even when appropriately placed. This pressure may also contribute to VP shunt displacement by encouraging extrusion of the shunt. Additionally, obese patients have significantly enlarged abdominal dead spaces into which the VP shunt can migrate, making shunt displacement difficult to detect [8]. One author posits that the abdominal fat pad/pannus shifting with patient activity may act as a windlass or pulley system; with upright positioning, the fat pad pulls the VP shunt caudad, and with sitting or lying, the fat pad shifts craniad, creating redundancy within the tubing and coiling it within the abdominal wall [11].

It seems intuitive that a solution to this migration problem might involve the neurosurgeon “tacking down” the VP shunt at the peritoneal entry point [12]. However, there are problems with simply fixing the VP shunt permanently in place. Unfortunately, the rate of shunt failure requiring shunt removal or revision is high; Rosenberg et al. estimated the need for revision to be about 2.5 revisions per patient, with the initial failure occurring at an average of 8.9 months after the initial VP shunt placement [13]. These failed shunts can typically be removed in a single motion through the matured shunt tract if the shunt is not fixed in place. Since removing VP shunts does occur with some frequency, it would be a disservice to the patient to permanently insert the device as part of a standard protocol when the only means to remove it is extremely complicated and challenging.

IIH patients with shunt dislodgement will typically present with recurrence of their headaches or vision loss, but they may present with isolated abdominal complaints, such as in our patient. CT scan of the head in these patients is often unremarkable, as they do not typically have enlarged ventricles. Clinical assessment of the abdomen may be inhibited by the patient's habitus. Therefore, a low threshold should be maintained for obtaining a shunt series or even an abdominal CT scan in obese patients with VP shunts complaining of abdominal pain or headache.

## 4. Conclusion

Shunt dislodgement, migration, and subsequent failure are common in obese patients who have shunts placed for IIH. The medical provider should maintain a high index of suspicion for shunt malfunction in these patients, particularly because clinical evaluation may be challenging due to habitus.
